# Identification, biological characteristics, and screening of control agents against root rot of *Ardisia crenata* Sims in China

**DOI:** 10.3389/fmicb.2025.1618299

**Published:** 2025-09-25

**Authors:** Zhien Xiao, Tiantian Guo, Yuxin Liu, Yue Yuan, Shaotian Zhang, Lu Liu, Shiying He, Yanling Long, Fuqiang Yin, Ming Liu

**Affiliations:** ^1^College of Biological and Food Engineering, Chongqing Three Gorges University, Chongqing, China; ^2^Chongqing Three Gorges Vocational College, College of Agriculture and Forestry Science and Technology, Chongqing, China; ^3^The Chongqing Engineering Laboratory for Green Cultivation and Deep Processing of the Three Gorges Reservoir Area’s Medicinal Herbs, Chongqing, China

**Keywords:** *Ardisia crenata* Sims, *Fusarium cugenangense*, root rot, biological characteristics, pesticides

## Abstract

*Ardisia crenata* Sims is included in the *Chinese Pharmacopoeia* and is a valuable traditional medicinal plant. *A. crenata* Sims root rot was first detected in 2023 in Kaizhou District, Chongqing City, China. This study was undertaken to elucidate the causal pathogen of *A. crenata* Sims root rot and the biological characteristics of the pathogen and to screen effective pesticides for its management. A pathogenic fungus was isolated and purified from typical lesions on *A. crenata* Sims, and it was identified using morphological and multi-gene combined analysis. The biological characteristics of the pathogen were investigated using the cross-inoculation method and spore counting technique, while the sensitivity of the pathogen to six chemical pesticides and six biological pesticides was evaluated using the growth rate method. The combination of morphological characteristics and polygenic association identified the pathogenic fungus as *Fusarium cugenangense,* a member of the *F. oxysporum* species complex. This is the first report of *F. cugenangense* causing root rot disease in *A. crenata* Sims. Among the six tested chemical pesticides, the 500 g/L fluazinam suspension concentrate (SC) showed the best inhibitory effect, with a half-maximal effective concentration (EC_50_) value of 0.129 μg/mL. Among the six tested biological pesticides, 3% zhongshengmycin soluble liquid (SL) showed the best inhibitory effect, with an EC_50_ value of 14.474 μg/mL. The conclusion is expected to lay a scientific basis for the diagnosis and control of *A. crenata* Sims root rot caused by *F. cugenangense*.

## 1 Introduction

*Ardisia crenata* Sims belongs to the *Ardisia* genus within the Myrsinaceae family and is widely distributed throughout the Yangtze River region and southern China, including Chongqing, Sichuan, Guizhou, Fujian, and Taiwan (Hu et al., 2020). *A. crenata* Sims was first documented in the *Compendium* of *Materia Medica* (Chen, 1990) and is included in the 2020 edition of the *Chinese Pharmacopoeia* (China Medical Science and Technology Press, 2020). In China, the roots of *A. crenata* Sims are used as the renowned traditional Chinese medicine known as “Zhushagen” (Liu et al., 2011; Tao et al., 2022). It mainly contains coumarin compounds, saponins, flavonoids, volatile oils, cyclic decapeptides, and phenolic compounds, among other chemical components (Ren et al., 2024). It is extensively utilized in the treatment of respiratory infections, toothaches, arthralgia, menstrual irregularities, and fertility regulation (Liu et al., 2011; Zheng et al., 2008).

Root rot, a severe plant disease with global impact, was first documented in Virginia apples (*Malus pumila*) in 1917 (Fromme and Thomas, 1917). It can be caused by diverse pathogens, including species of *Fusarium*, *Ilyonectria* (Li et al., 2025), *Phytophthora*, *Armillariella mellea* (Wang et al., 2025), *Corynespora cassiicola* (Luo et al., 2025), *Boeremia exigua* (Liu et al., 2025), *Phytophthora cryptogea*, and *Macrophomina phaseolina* (Li et al., 2024). From 2023 to 2024, root rot of unknown etiology was observed in *A. crenata* Sims plantations in Kaizhou District, Chongqing, with a disease prevalence of 25–35%. This disease severely compromises the yield and medicinal value of *A. crenata* Sims by damaging its roots.

*Fusarium* exhibits substantial genetic variation and remarkable species diversity. The identification of *Fusarium* species is relatively complex, and conventional morphological diagnosis is unreliable (Thomas et al., 2019). Therefore, molecular biology identification has become an important tool for the accurate diagnosis of *Fusarium* species. Researchers usually use the translation elongation factor 1-alpha (*TEF1*) gene (Zheng et al., 2023) and the RNA polymerase II second largest subunit genes (*RPB1* and *RPB2, respectively*) (O’Donnell et al., 2021) for the molecular identification of *Fusarium* species.

In this study, roots from typical symptomatic plants of *A. crenata* Sims were collected for the isolation, purification, and pathogenicity determination of pathogenic fungi. The pathogenic fungi were identified based on morphological characteristics and multi-gene sequence analysis of the *TEF1*, *RPB1,* and *RPB2* loci. Based on this, the biological properties of the pathogenic fungi were studied and the screening of control agents was carried out to provide a scientific basis for the precise management of root rot.

## 2 Materials and methods

### 2.1 Disease investigation and collection

The disease incidence in *A. crenata* Sims plantations in Kaizhou District, Chongqing City (31°34′11″N, 108°25′23″E), was assessed using the five-point sampling method. A total of 12 samples were collected from the plantations for the isolation of pathogenic fungi. The disease incidence (Parmar et al., 2015) was calculated using the following formula:


$$\text{Disease Incidence}(\%)=\frac{\text{Number of diseased plants}}{\text{Total number of plants}}X100\%$$

### 2.2 Experimental methods

#### 2.2.1 Pathogenic fungal isolation and purification

The collected samples were rinsed under tap water for 10–15 min. After naturally drying the roots, disease samples measuring 3 mm × 3 mm from the junction of diseased and healthy tissue were cut with a sterile scalpel. The pieces were disinfected in 75% ethanol for 1 min and in 3% NaClO for 6 min, then they were rinsed with sterile water 3 times for 30 s each time (Wang et al., 2021). After drying on sterile filter paper, the samples were inoculated onto potato dextrose agar (PDA) and incubated for 5 days at 25 °C. After the appearance of fungal colonies, blocks of tissue were removed from the edges of the colonies for purification. The purified colonies were then examined and documented for their morphological traits, including color, size, and shape (Chang et al., 2020). The purified fungal isolates were stored at 4 °C for subsequent studies.

#### 2.2.2 Pathogenicity tests

Representative isolates were selected for pathogenicity testing on *A. crenata* Sims roots using the spore suspension method (Fu et al., 2019). Healthy, intact roots of *A. crenata* Sims were collected and surface-disinfected with 75% ethanol, followed by rinsing with sterile water. A spore suspension (1 × 10^6^ spores/mL) was prepared using 1 mL of Tween, 3 mL of glycerol, and 100 mL of sterile water. A 0.5-cm-long wound was created on the root surface using a sterile scalpel, and the spore suspension was uniformly applied to the wound. A suspension without added pathogens served as a blank control. The inoculated roots were placed in a preservation box with a double layer of filter paper (soaked in sterile water) for easy observation. A total of three replicates were set up for each treatment.

The inoculated samples were placed in a constant temperature and humidity incubator and cultivated for 14 days under conditions of 28 °C and 90% relative humidity (RH). When these plants developed root rot disease again, tissue isolation was conducted from the affected regions. If the re-isolated strain was identical to the originally inoculated strain, Koch’s postulates were thereby fulfilled.

#### 2.2.3 Morphology observation of the pathogenic fungi

The purified strains were inoculated onto PDA and carnation leaf agar (CLA) media and incubated at a constant temperature of 28 °C for 6 days. The colony color and morphology were observed. Mycelia were collected and examined microscopically to observe spore morphology and measure spore size (*n* = 50; Loganathan et al., 2020).

#### 2.2.4 Primer design and synthesis

Based on the *RPB1* gene sequence of the *Fusarium oxysporum* species complex (FOSC) published in the NCBI database, a pair of specific primers was designed, namely the forward primer *RPB1*-YF and the reverse primer *RPB1*-XR. Primer sets EF728/EF986 (Carbone and Kohn, 1999) were used to amplify the *TEF1* gene and primer sets 5F2/7CR (Reeb et al., 2004) were used to amplify the *RPB2* gene. A total of three gene primers were synthesized by Sangon Biotech (Shanghai) Co., Ltd. (Chengdu).

#### 2.2.5 Molecular identification of the pathogenic fungi

A total of three representative strains ZSG4, ZSG18, and ZSG29 were selected and cultivated on the PDA medium at 25 °C for 10 days. Genomic DNA was extracted from the mycelia using a commercial DNA extraction kit (CW0553S, Jiangsu Kangwei Century Biotechnology Co., Ltd.) according to the manufacturer’s instructions. After passing the test, the extracted DNA was stored at −20 °C (Guo et al., 2000).

The extracted DNA was subjected to polymerase chain reaction (PCR) amplification targeting partial regions of the three genes, namely, *TEF1*, *RPB1*, and *RPB2*, which were amplified with primers EF728/EF986, YF/XR, and 5F2/7CR, respectively (Table 1).

**Table 1:** Gene markers and primer pairs used in this study of *F. cugenangense* ZSG4, ZSG18, and ZSG29.

Locus	Primer	Sequences of primers 5′–3′	PCR amplification procedures	Manufacturer
*TEF1*	*EF728*	CATCGAGAAGTTCGAGAAGG	94 °C 90 s; 35 cycles of 94 °C 45 s, 55 °C 45 s, 72 °C 1 min; 72 °C 10 min;	Sangon Biotech (Shanghai) Co. Ltd. (Chengdu)
	*EF986*	TACTTGAAGGAACCCTTACC		
*RPB1*	*YF*	TTCCTCACAAAGGAGCAAATCA	95 °C 5 min; 35 cycles of 95 °C 31 s, 55 °C 31 s, 72 °C 44 s; 72 °C 10 min;	Sangon Biotech (Shanghai) Co. Ltd. (Chengdu)
	*XR*	TAAAGACTCGCTTAGCATACTCG		
*RPB2*	*5F2*	GGGGWGAYCAGAAGAAGGC	94 °C 5 min; 35 cycles of 94 °C 60 s, 55 °C 31 s, 72 °C 90s; 72 °C 10 min;	Sangon Biotech (Shanghai) Co. Ltd. (Chengdu)
	*7CR*	CCCATRGCTTGYTTRCCCAT		

The total volume of the PCR mixture was 25 μL, containing 12.5 μL of Taq DNA polymerase mix, 0.5 μL each of forward and reverse primers, 2 μL of DNA template, and 9.5 μL of distilled water (ddH_2_O). After amplification, part of the PCR products was detected by 1% agarose gel electrophoresis. The remaining reactants were sent to Sangon Biotech (Shanghai) Co., Ltd. (Chengdu) for sequencing. The obtained sequences were compared and analyzed using the Fusarioid-ID database,^1^ and the sequences of all FOSC strains were downloaded (Table 2). *Myrothecium cinctum* ATCC 22270 was used as the outgroup. The PhyloSuite software (Xiang et al., 2023) was used to assemble single-gene sequences into concatenated sequences. A phylogenetic tree was constructed based on the concatenated sequences (*TEF1*, *RPB1*, and *RPB2*) using the neighbor-joining algorithm in MEGA 11 (Tamura et al., 2021).

**Table 2:** GenBank and Fusarioid-ID accession numbers used in the phylogenetic tree.

Species	Strain	Data bank	*TEF1*	*RPB1*	*RPB2*
*Fusarium cugenangense*	ZSG4	NCBI	PQ476181	PX126198	PX108291
*Fusarium cugenangense*	ZSG18	NCBI	PQ476182	PX126199	PX108292
*Fusarium cugenangense*	ZSG29	NCBI	PQ476183	PX126200	PX108293
*Fusarium cugenangense*	CBS 102029	Fusarioid-ID	LS479669	LS479477	LS479221
*Fusarium cili*	GUCC 190024 1	Fusarioid-ID	OR043885	OR043785	OR043830
*Fusarium cili*	GUCC 190024 2	Fusarioid-ID	OR043886	OR043786	OR043831
*Fusarium phialophorum*	InaCC F826	Fusarioid-ID	LS479700	LS479505	LS479251
*Fusarium phialophorum*	InaCC F995	Fusarioid-ID	OP487296	LS479570	LS479318
*Fusarium odoratissimum*	InaCC F822	Fusarioid-ID	LS479828	LS479618	LS479386
*Fusarium odoratissimum*	InaCC F823	Fusarioid-ID	LS479838	LS479628	LS479398
*Fusarium rosae roxburghii*	GUCC 190111 1	Fusarioid-ID	OR043910	OR043800	OR043853
*Fusarium rosae roxburghii*	GUCC 190111 2	Fusarioid-ID	OR043911	OR043801	OR043854
*Fusarium thapsinum*	CBS 135921	Fusarioid-ID	MW402057	MW402583	MW402800
*Fusarium thapsinum*	CBS 100313	Fusarioid-ID	MW401962	MW402495	MW402781
*Fusarium lacertarum*	LC7927	Fusarioid-ID	MK289637	MK289866	MK289791
*Fusarium lacertarum*	LLC3165	Fusarioid-ID	OP487182	OP486351	OP486751
*Myrothecium cinctum*	ATCC 22270	NCBI	AY489605.1	AY489638.1	EF692512.1

#### 2.2.6 Biological experiments

Based on the above experiments, the strain ZSG18 exhibited the strongest pathogenicity. Therefore, we selected the strain ZSG18 for further in-depth study. The effects of different culture media, including PDA, bean agar (BA), carrot agar (CA), potato sucrose agar (PSA), corn meal agar (CMA), Czapek dox agar (CDA), oatmeal agar (OA), and water agar (WA), as well as temperatures (5 to 35 °C), pH values (5.0 to 12.0), photoperiods (24-h darkness, a 12-h light/dark cycle, and 24-h light), carbon sources, and nitrogen sources, on colony growth and spore production of the pathogenic fungus were studied.

Czapek agar medium served as the basal medium. Equal amounts of glucose, lactose, fructose, malt dust, mannitol, and soluble starch were used to substitute sucrose as the carbon source, with carbon source-free Czapek agar medium serving as the control. Similarly, equal amounts of peptone, glycine, beef extract, yeast extract powder, ammonium chloride (NH_4_CL), and urea were utilized to replace sodium nitrate (NaNO_3_) as the nitrogen source, with nitrogen-free Czapek agar medium as the control.

The strains were cultivated and propagated for 144 h in a 28 °C incubator, under the conditions previously stated. The diameter of colony growth was measured using the crossover method, serving as an indicator of mycelial growth. In addition, the sporulation of the colonies under each culture condition was measured using a blood count plate via microscopic observation. Each treatment was replicated three times.

The mycelial plugs (5 mm) were picked and placed into a sterilized centrifuge tube containing 2 mL of sterile water. The tubes were then placed in a constant temperature water bath preheated to temperatures ranging from 40 to 65 °C (with a gradient of 5 °C) for 10 min (preheated for 1 min). The mycelial plugs were removed and cooled to room temperature. The treated mycelial plugs were placed in the center of PDA medium plates and put into an incubator at 28 °C. Each treatment was repeated three times. The growth status of the treated samples was observed continuously for 6 days, and the lethal temperature range was determined.

#### 2.2.7 Fungicide assays

The antifungal activities of six chemical pesticides and six biological pesticides against the strain ZSG18 were evaluated using the mycelial growth rate method (Xin et al., 2020). After diluting each of these pesticides (Tables 3, 4), they were added to the PDA culture medium and thoroughly mixed to create the treatment group. PDA medium without pesticides was used as the blank control. To ensure the reliability and reproducibility of the results, there were three replicates per pesticide concentration. The inoculated plates were incubated in a constant temperature incubator at 28 °C for 6 days. Colony diameter (mm) was measured using the crisscross method. The percent inhibition of mycelial growth (PIMG) was calculated using the following formula, where F is the diameter of the fungal plug, C represents the control colony diameter, and T represents the treatment colony diameter (Muhammad et al., 2021);


$$\text{Antifungalrate}(\%)=\frac{C-T}{C-F}X100\%$$

**Table 3:** The names and source agents of the six chemical pesticides tested on *F. cugenangense* ZSG18.

Fungicide name	Active ingredient quality fraction/%	Type	Manufacturer	Substance concentration (μg/mL)
Fluazinam	500 g/L	SC	Shandong Zouping Pesticide Co., Ltd.	0.5, 0.1, 0.05, 0.01, 0.001
Difenoconazole	40%	SC	Shandong Baixin Biotechnology Co., Ltd.	12.8, 3.2, 0.2, 0.05, 0.0125
Tebuconazole	25%	WP	Yancheng Shuangning Agro-Chemical Co., Ltd.	12.8, 3.2, 0.8, 0.2, 0.05
Flusilazole	10%	EW	Jiangxi Heyi Chemical Co., Ltd.	2, 1, 0.5, 0.25, 0.0625
Pyraclostrobine	30%	SC	Qingdao Haina Biotechnology Co., Ltd.	3.2, 0.8, 0.4, 0.2, 0.1
Imazalil	20%	EW	Yifan Biotechnology Group Co., Ltd	4.8, 1.2, 0.6, 0.3, 0.15

SC is suspension concentrate, WP is wettable powder, EW is emulsion in water.

**Table 4:** The names and source agents of the six biological pesticides tested on *F. cugenangense* ZSG18.

Active ingredient	Mechanism of action	Manufacturer	Substance concentration (μg/mL)
Zhongshengmycin 3% (SL)	Interference with fungal cell wall synthesis and rupture of fungal cell membranes	Fujian Kaili Biological Products Co., Ltd	150, 100, 66.6, 8.7, 5.8
Cnidii Fructus 1% (EW)	Interferes with cell wall and cell membrane, inhibits sugar metabolism, and affects spore germination and mycelial growth	Inner Mongolia Qingyuanbao Biotechnology Co., Ltd	40, 20, 2.5, 1.25, 0.625
Ethylicin 80% (EC)	Interferes with cell membrane and cell wall synthesis and inhibits fungal growth	Mengzhou Guangnong Huize Biotechnology Co., Ltd	60, 30, 15, 7.5, 3.75
Avermectin 5% (EC)	Interference with fungal cell wall chitin synthesis and alteration of cell membrane permeability	Shandong Libang Agrochemical Co., Ltd	160, 80, 20, 10, 5
Berberine 4% (AS)	Disrupts cell membranes and inhibits cell walls	Inner Mongolia Qingyuanbao Biotechnology Co., Ltd	500, 250, 62.5, 31.2, 15.625
Ningnanmycin 8% (AS)	Inhibits fungal enzyme activity	Deqiang Biotechnology Co., Ltd	2000, 250, 125, 62.5, 31.25

SL is soluble liquid, EW is emulsion in water, EC is emulsifiable concentrate, AS is aqueous solution.

Microsoft Excel 2021 (Brian et al., 2024) was utilized to calculate the toxicity regression equation, where x represents the logarithm of the fungicide concentration in μg/mL and y represents the inhibition rate, and the correlation coefficient for each fungicide. The SPSS 26.0 software (Tan et al., 2021) (IBM Corp., United States) was employed to determine the half-maximal effective concentration (EC_50_) value for each fungicide (Dou et al., 2023). EC_50_ values were determined using probit regression analysis in SPSS 26.0, with inhibition rate as the dependent variable and concentration as the independent variable, using a logit model for transformation. The EC_50_ value was derived as the concentration corresponding to a probability output of 0.5.

#### 2.2.8 Statistical analysis

The analysis of the data was conducted using one-way analysis of variance and the least significant difference test in the SPSS 26.0 software, and the Origin software (Li et al., 2024) was used to create the figures.

## 3 Results

### 3.1 Natural symptoms

**Figure 1:**
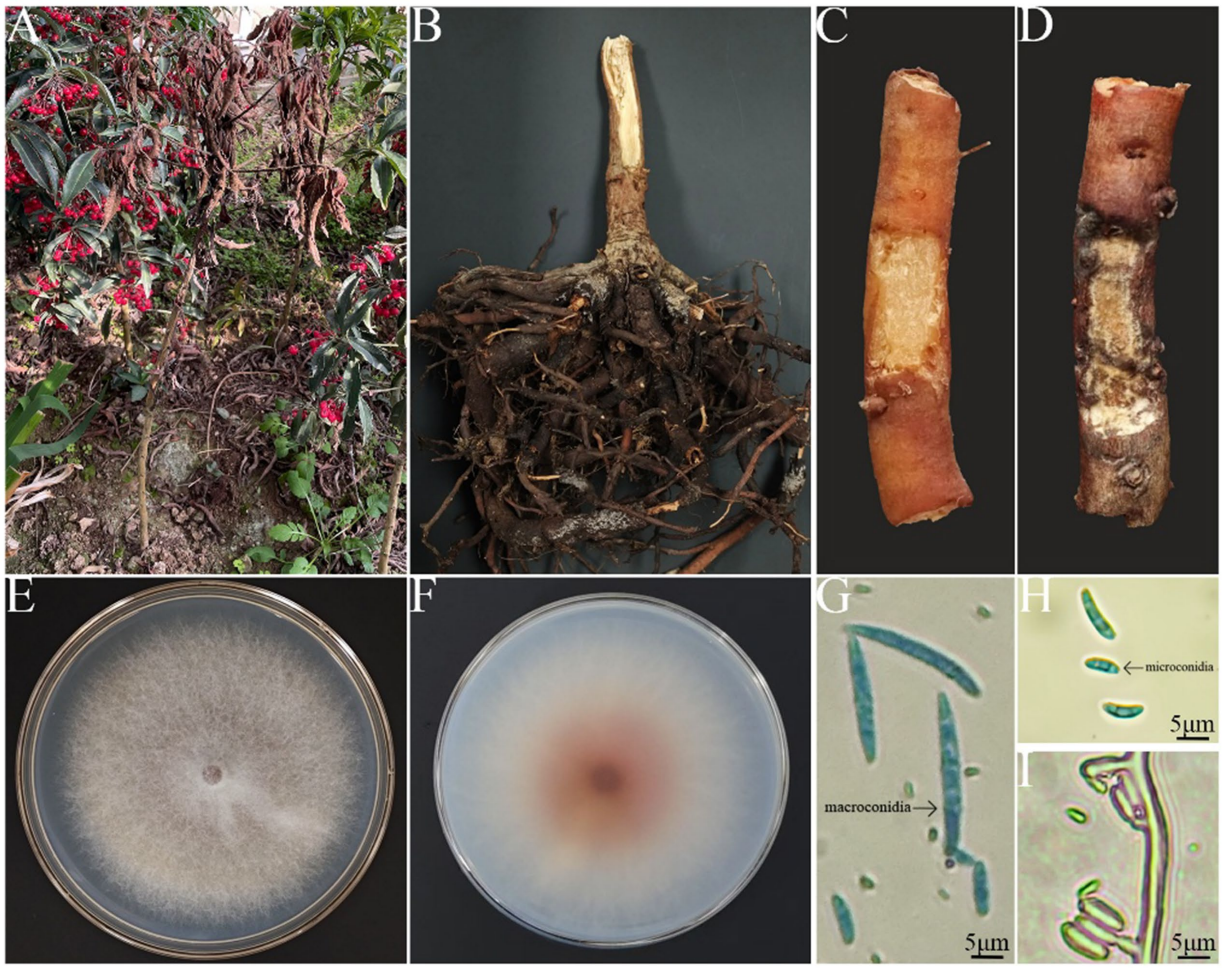
Symptoms of root rot caused by *Fusarium* spp. **(A)** Disease symptoms in the field. **(B)** Root status of the diseased plants. **(C)** Cross-sections of the roots from the control group *A. crenata* Sims. **(D)** Cross-sections of the roots from *A. crenata* Sims inoculated with the strain. **(E)** Colony on PDA (front view). **(F)** Colony on PDA (back view). **(G)** The morphology and size of macroconidia. **(H)** The morphology and size of microconidia. **(I)** Conidiophores.

Root rot in *A. crenata* Sims typically begins between March and April, peaking between July and September. The incidence is relatively low from October to November, ranging from 25 to 35%. After disease onset, roots become blackened, softened, and rotted. The root cortex separates from the root pith, losing the ability to absorb water and nutrients. The leaves gradually wilt and the fruits fall off (Figures 1A,B). Eventually, this leads to the whole plant withering and dying. However, the stem does not turn black and rot.

### 3.2 Morphological identification of the isolates

The colony morphology of the isolate appeared white when it was growing on PDA and looked short and furry (Figures 1E,F). The back of the colony center was purplish red, with edges that were white. On the CLA medium, the pathogen’s macroconidia were abundant in number, ranging from falcate to almost straight in shape, measuring (24.75 ~ 46.25) μm × (3.75 ~ 4.85) μm, and containing 3–5 septa (Figure 1G). On the PDA medium, the pathogen’s microconidia were either oval or kidney-shaped, measuring (5.7 ~ 11.74) μm × (2.34 ~ 3.96) μm, and had 0–3 septa (Figure 1H). Conidiogenous cells were monophialidic, borne on sporodochia or aerial hyphae, or arising directly from the hyphae as lateral phialides (Figure 1I).

### 3.3 Pathogenicity test

The symptoms of *A. crenata* Sims root infection following inoculation with the pathogen were consistent with those observed in the field (Figure 1D). In contrast, the control group did not show any signs of the disease (Figure 1C). Ultimately, through re-isolation, fungal isolates were obtained that resembled the morphology of the original inoculum, thereby confirming Koch’s postulates. This indicates that the inoculated strain was indeed the pathogen responsible for the infection in the *A. crenata* Sims root.

### 3.4 Molecular identification of the pathogen

The sequences of *TEF1* (GenBank accession numbers PQ476181, PQ476182, and PQ476183), *RPB1* (accession numbers PX126198, PX126199, and PX126200), and *RPB2* (accession numbers PX108291, PX108292, and PX108293) amplified by PCR in the present study have been deposited in the NCBI database.

Through comparative analysis of strains ZSG4, ZSG18, and ZSG29 using the Fusarioid-ID database, the *TEF1* gene sequence of the strain ZSG4 exhibited 95.49% similarity to *F. cugenangense* InaCC F984 (Fusarioid ID: LS479757), while the *RPB1* and *RPB2* genes demonstrated 100 and 99.89% similarity to *F. cugenangense* CBS 130308 (Fusarioid ID: HM347143) and CBS 102029 (Fusarioid ID: LS479221), respectively. For the strain ZSG18, the *TEF1*, *RPB1*, and *RPB2* genes exhibited 99.18, 99.67, and 99.88% similarity, respectively, to the corresponding genes of *F. cugenangense* CBS 130308 (MH485011 and HM347143) and CBS 102029 (LS479221). The strain ZSG29 demonstrated 98.02% (*TEF1*), 99.92% (*RPB1*), and 99.88% (*RPB2*) similarity to the corresponding genes of *F. cugenangense* CBS 130308 (MH485011 and HM347143) and CBS 102029 (LS479221).

**Figure 2:**
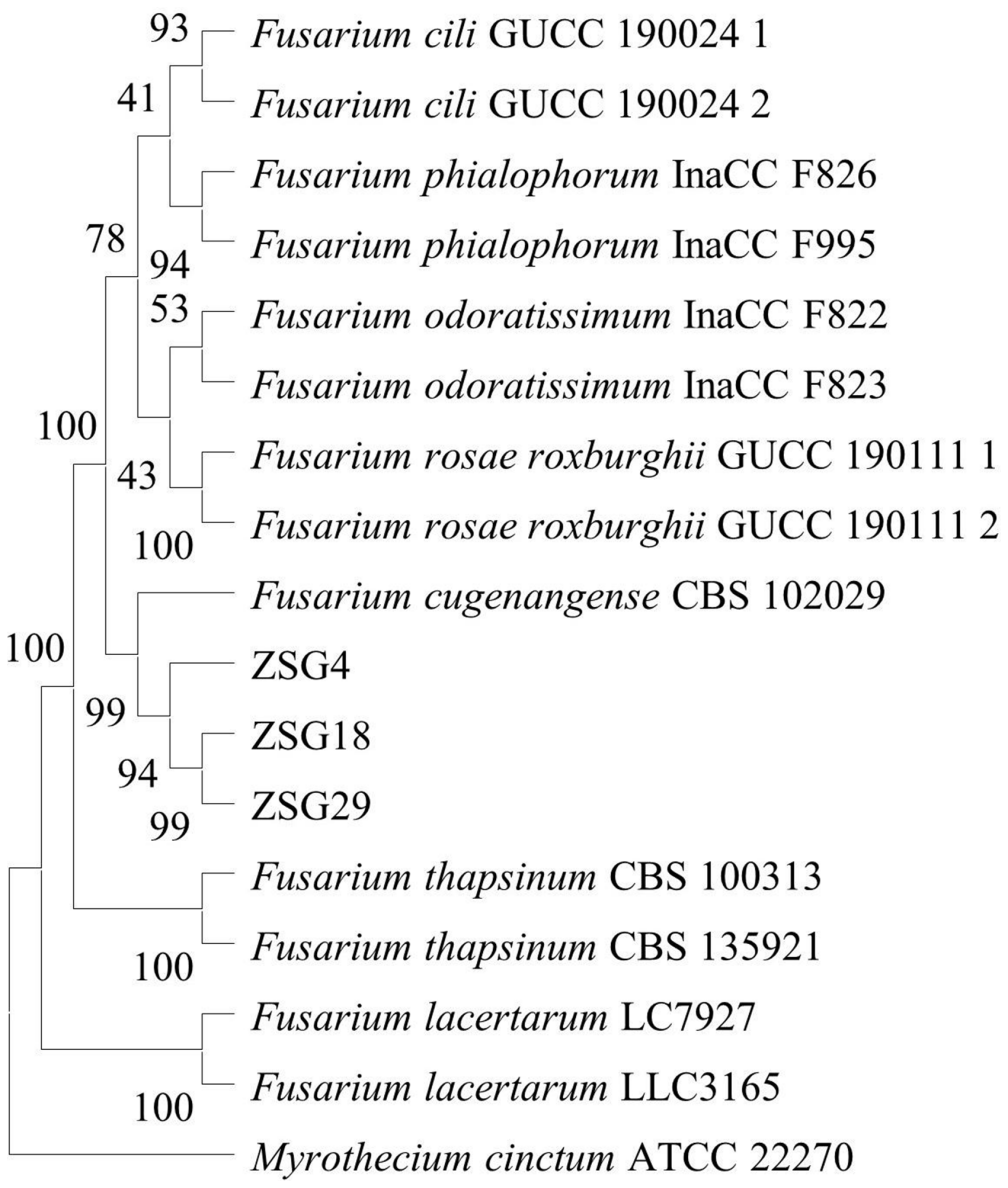
Phylogenetic tree inferred from concatenated *TEF1*, *RPB1,* and *RPB2* sequences using the neighbor-joining method. Bootstrap values (%) presented at the branches were calculated from 1,000 replications. Values below 50% are hidden. The scale bar indicates a 5% sequence difference.

Combined with the *TEF1*, *RPB1,* and *RPB2* sequences derived from this study, a phylogenetic tree was constructed using the neighbor-joining method, incorporating representative isolates from 14 related species to determine phylogenetic relationships. The results showed that isolates ZSG4, ZSG18, and ZSG29, along with the *Fusarium cugenangense* CBS 102029, clustered within the same branch, with a bootstrap support value of 99%. Therefore, the pathogen was identified as *F. cugenangense* (Figure 2), as evidenced by both morphological and molecular evidence.

### 3.5 The biological characteristics of *Fusarium cugenangense* ZSG18

**Figure 3:**
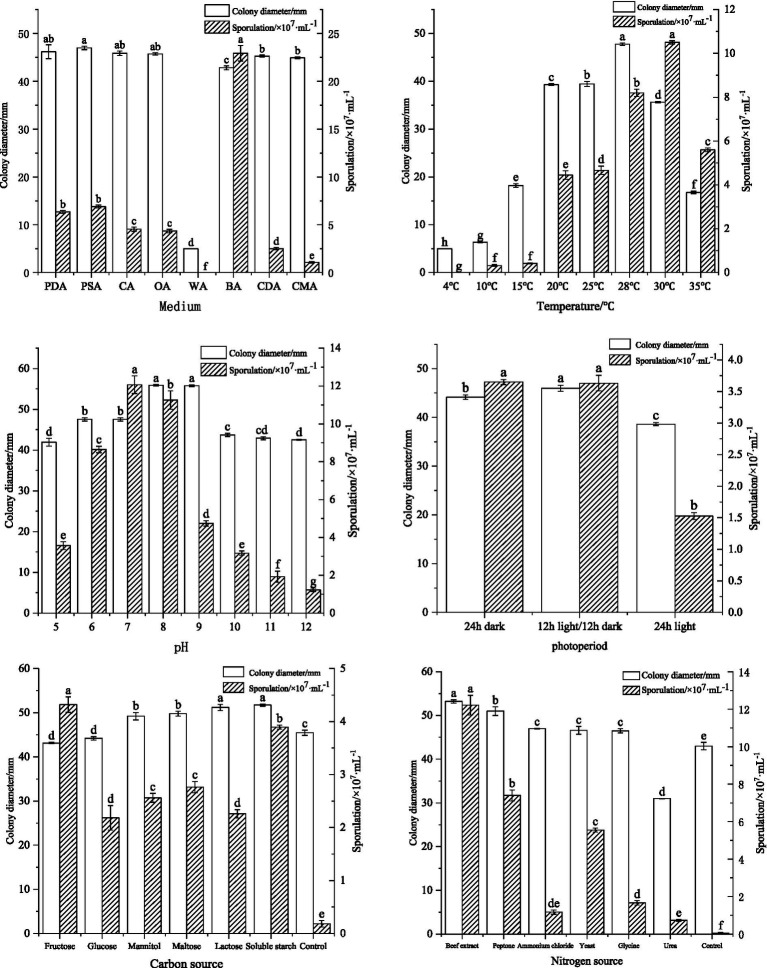
Effects of culture media, temperature, pH, photoperiod, carbon source, and nitrogen source on the mycelial growth and sporulation of *F. cugenangense* ZSG18. Different lowercase letters represent significant differences (*p* < 0.05). Data are presented as mean ± SE.

*F. cugenangense* ZSG18 exhibited the fastest mycelial growth on PSA, PDA, CA, and OA media. On the BA medium, it produced the highest number of spores, with significant differences compared to other media. When cultivated on the WA medium, the mycelium did not grow and no spores were produced. (Figure 3). Optimal mycelial growth occurred at 28 °C, while optimal spore production was observed at 30 °C. No growth or spore production occurred at 4 °C, with significant differences compared to other temperatures (Figure 3). At all pH values tested, the pathogen grew and sporulated. Mycelial growth was fastest at pH values of 8.0 and 9.0. Spore production was optimal at pH 7.0, and the differences were significant compared to other pH values (Figure 3). Mycelial growth was best under a 12-h light/dark cycle, which was significantly different from other photoperiods. Spore production was optimal under a 12-h light/dark cycle and 24-h darkness (Figure 3). *F. cugenangense* showed the fastest mycelial growth on soluble starch and lactose. Spore production was highest on fructose, and these differences were significant compared to other carbon sources (Figure 3). Although the colonies grown on the Czapek medium without a carbon source were large in diameter, the mycelium was particularly sparse. *F. cugenangense* exhibited optimal mycelial growth and spore production when beef extract served as the nitrogen source, and the differences were significant compared to other nitrogen sources (Figure 3). Although the colonies grown on the Czapek medium without a nitrogen source were large in diameter, the mycelium was particularly sparse.

**Figure 4:**
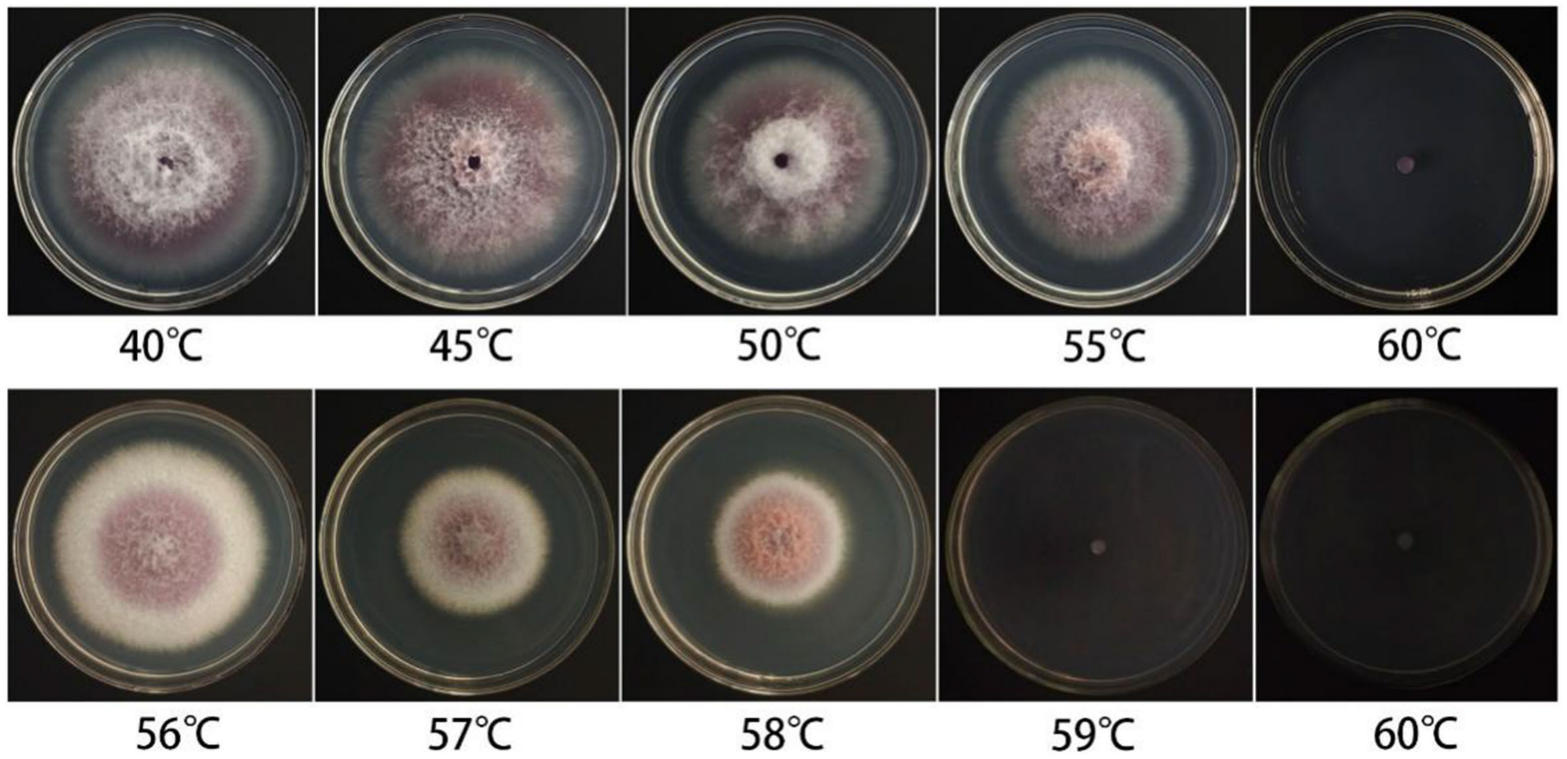
Lethal temperature test of *F. cugenangense* ZSG18.

The fungal cake of *F. cugenangense* ZSG18 was subjected to water baths at varying temperatures for experimentation. The fungal cake grew and produced colonies when soaked in water at temperatures ranging from 35 to 55 °C for 10 min. To further clarify the lethal temperature, a water bath treatment starting at 55 °C with a 1 °C incremental gradient was applied. After a 10-min treatment in a water bath at ≥59 °C, the fungal cake in the medium no longer grew. The results showed that the lethal temperature of this pathogenic fungus is 59 °C (Figure 4).

### 3.6 Screening of six chemical pesticides under indoor conditions

**Figure 5:**
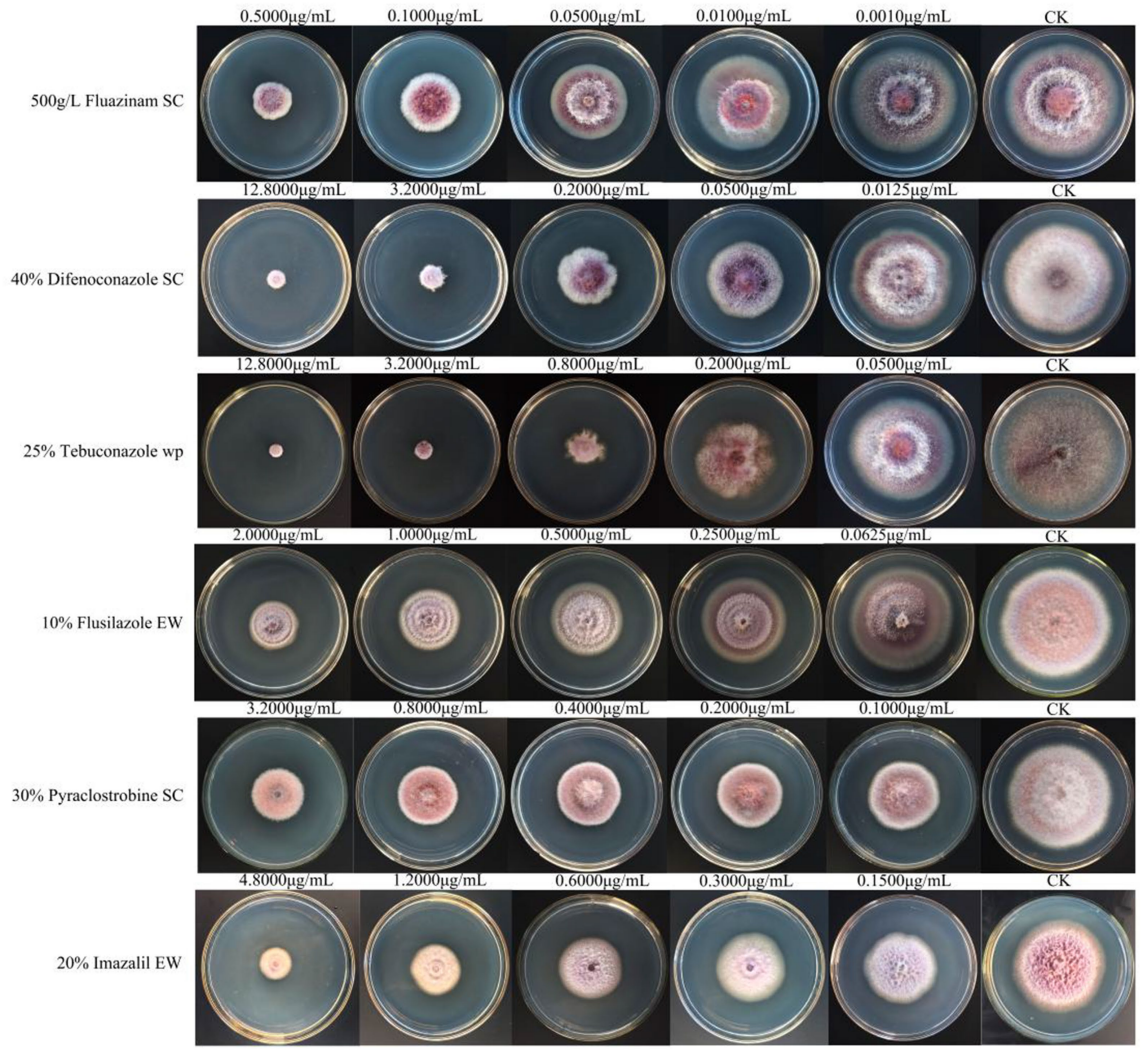
The effects of the six chemical pesticides on the mycelial growth and spore production of *F. cugenangense* ZSG18.

Among the six chemical pesticides tested (Figure 5), the EC_50_ values were as follows: 20% imazalil emulsion in water (EW) (EC_50_, 1.409 μg/mL) > 30% pyraclostrobin suspension concentrate (SC) (EC_50_, 0.853 μg/mL) > 10% flusilazole EW (EC_50_, 0.753 μg/mL) > 25% tebuconazole wettable powder (WP) (EC_50_, 0.239 μg/mL) > 40% difenoconazole SC (EC_50_, 0.193 μg/mL) > 500 g/L fluazinam SC (EC_50_, 0.129 μg/mL). Among them, the 500 g/L fluazinam SC had the strongest inhibitory effect on *A. crenata* Sims root rot, with an EC_50_ of 0.129 μg/mL (Table 5).

**Table 5:** The inhibitory effects of the six chemical pesticides on *F. cugenangense* ZSG18.

Fungicide	Toxic regression equation	EC_50_ (μg/mL)	Correlation coefficient (R^2^)	95% confidence intervals
500 g/L Fluazinam SC	Y = 5.7723 + 0.3783X	0.129	0.9976	0.106–0.162
40% Difenoconazole SC	Y = 5.5652 + 0.3452X	0.193	0.997	0.156–0.238
25% Tebuconazole WP	Y = 0.8554 + 0.5935X	0.239	0.9959	0.205–0.275
10% Flusilazole EW	Y = 5.1139 + 0.4016X	0.753	0.9995	0.635–0.909
30% Pyraclostrobine SC	Y = 5.0197 + 0.1241X	0.853	0.9944	0.509–1.890
20% Imazalil EW	Y = 4.8571 + 0.4197X	1.409	0.9902	1.186–1.714

### 3.7 Screening of six biological pesticides under indoor conditions

**Figure 6:**
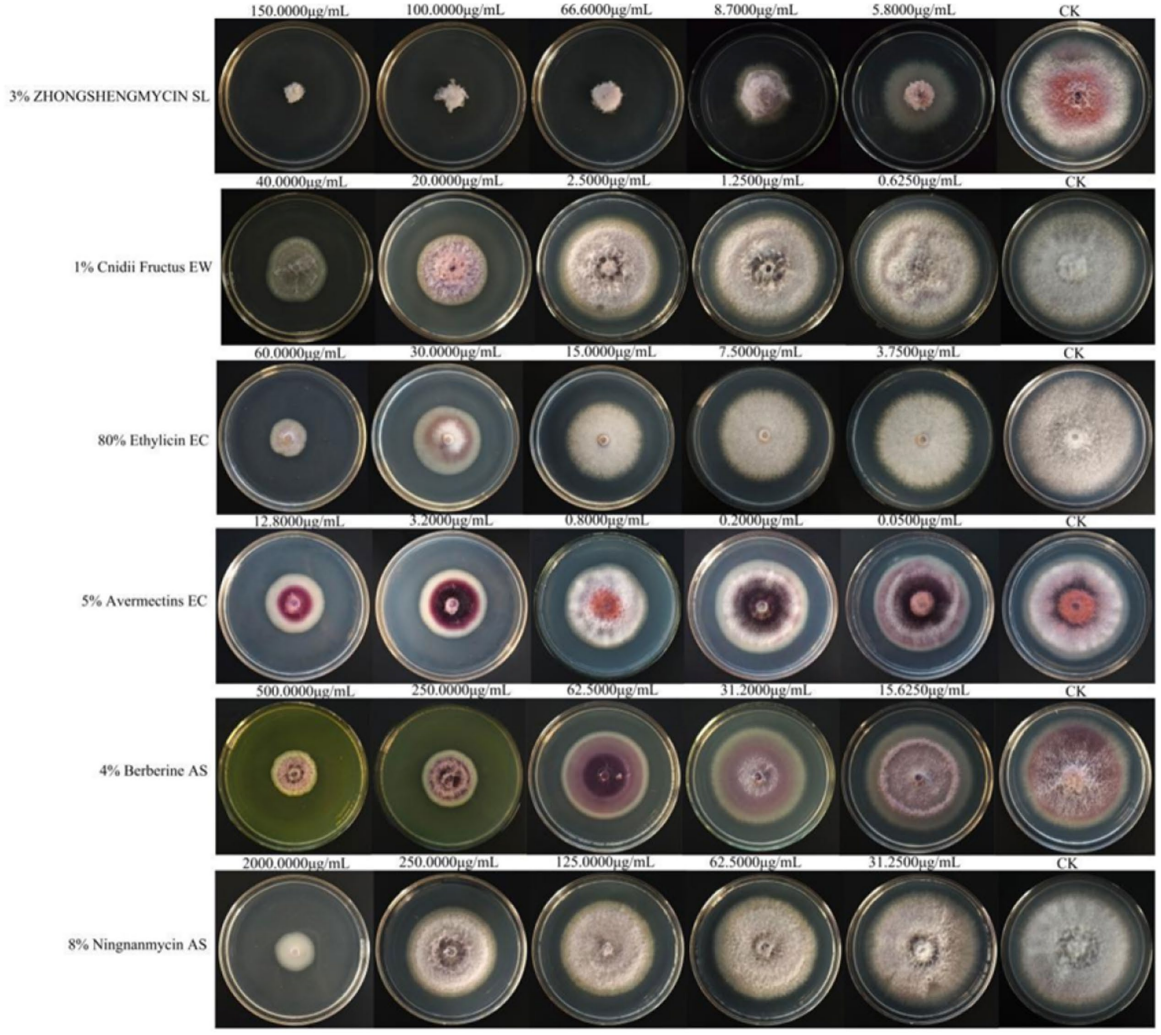
The effects of the six biological pesticides on the mycelial growth and spore production of *F. cugenangense* ZSG18.

Among the six biological pesticides tested (Figure 6), 3% zhongshengmycin soluble liquid (SL) had the most effective inhibitory effect, with an EC_50_ value of 14.474 μg/mL, followed by 1% Cnidii Fructus (EW) and 80% ethylicin emulsifiable concentrate (EC). The 8% ningnanmycin aqueous solution (AS) was the least effective, with an EC_50_ of 608.527 μg/mL (Table 6).

**Table 6:** The inhibitory effects of the six biological pesticides on *F. cugenangense* ZSG18.

Fungicide	Toxic regression equation	EC_50_ (μg/mL)	Correlation coefficient (R^2^)	95% confidence intervals
3% Zhongshengmycin SL	Y = 3.4861 + 0.5666X	14.474	0.9964	12.496–16.576
1% Cnidii Fructus EW	Y = 3.4182 + 0.4786X	27.357	0.9965	22.524–34.158
80% Ethylicin EC	Y = 2.5161 + 0.7392X	28.939	0.9903	26.136–32.364
5% Avermectin EC	Y = 3.0094 + 0.3916X	159.854	0.9962	124.059–219.500
4% Berberine AS	Y = 2.5005 + 0.487X	168.450	0.9919	144.957–198.619
8% Ningnanmycin AS	Y = 2.2571 + 0.4282X	608.527	0.9918	499.039–765.615

## 4 Discussion

This study conducted the isolation and identification of the pathogenic fungus causing the root rot disease in *A. crenata* Sims. A combined analysis of morphological characteristics and polygene sequencing identified the pathogen responsible for root rot in *A. crenata* Sims plantations in Kaizhou District, Chongqing, China, as *F. cugenangense*. This is the first report of *F. cugenangense* causing root rot disease in *A. crenata* Sims.

*Fusarium* spp., belonging to the order Hypocreales of the phylum Ascomycota, were first described in 1809 by Link (Aoki et al., 2014). The main pathogenic *Fusarium* spp. complexes include the *F. oxysporum* species complex (FOSC), *Fusarium graminearum* species complex (FGSC), and *Fusarium tricinctum* species complex (FTSC). Among these, the FOSC is the dominant species (Jiang et al., 2024). The FOSC, as a soil-borne plant pathogen, is listed as one of the “Top Ten Plant Pathogenic Fungi in the World” due to its strong pathogenicity and wide range of hosts (Dean et al., 2012). The FOSC includes *Fusarium cugenangense*, *Fusarium oxysporum*, *Fusarium nirenbergiae*, and *Fusarium lacertarum*, as well as multiple other cryptic species. Previous studies show that the FOSC can cause root rot (Shrestha et al., 2024), wilt (Aiello et al., 2021), stem rot (Yang et al., 2022), and so on. The FOSC accelerates plant infection by producing cell wall-degrading enzymes and toxins, and through signal transduction pathways, it rapidly enters the plant body to reproduce and cause disease (Liang et al., 2014).

The research indicates that pathogenic fungi from different host sources have differences in biological characteristics (Liu et al., 2017). In this study, the optimal mycelial growth temperature was 28 °C. The optimal mycelial sporulation temperature was 30 °C, the optimal spore production pH was 7.0, and the optimal spore production carbon source was fructose. The lethal temperature for the pathogenic fungi was 59 °C. Yang et al. (2025) isolated *F. cugenangense* from diseased *Camellia sinensis* plants and found that the optimal temperature for mycelial growth was 25 °C. The optimal mycelial sporulation pH was 9.0, and the optimal spore production carbon source was sucrose. These findings differ from the results of this study, which may be due to differences in the host environments. Notably, this study revealed a discrepancy between the optimal temperatures for mycelial growth and mycelial sporulation. This may be due to the fact that the growth of the mycelium and mycelial sporulation are related to nutrient uptake, adaptation to environmental conditions, and their own metabolic pathways. These results help us understand *F. cugenangense* root rot development and formulate effective control measures.

Among the six chemical pesticides evaluated for their inhibitory effects on mycelial growth, the 500 g/L fluazinam SC showed the strongest inhibitory activity against the root rot pathogen, with an EC_50_ value of 0.129 μg/mL. Fluazinam (FZN) is a preventative chemical pesticide from the pyridinamine group (Lee et al., 2012). Fluazinam inhibits all stages of the infection process by inhibiting spore germination, mycelial penetration, growth, and spore formation. It is the only mitochondrial oxidative phosphorylation uncoupler currently available (Song et al., 2024). In previous studies, fluazinam has been shown to be a good bacteriostatic agent against *Botrytis cinerea* (Qiao et al., 2013), *Alternaria alternata* (Bian et al., 2024), *Lasiodiplodia theobromae* (Zhang et al., 2025), *Macrophomina phaseolina* (Shen et al., 2025), *Cercospora citrullina* (Zeng et al., 2024), and others. Given the findings of the present study, fluazinam can be selected for its high feasibility in controlling root rot of *A. crenata* Sims caused by *F. cugenangense*.

The use of safe biopesticides in the cultivation of Chinese herbal medicines is a future development trend. In the present study, six kinds of biopesticides showed inhibitory effects on *F. cugenangense*, but the one with the strongest inhibitory effect on *F. cugenangense* was 3% zhongshengmycin SL, with an EC_50_ of 14.474 μg/mL. It is a new type of agricultural antibiotic produced by the fermentation of *Streptomyces violaceusniger*, belonging to the N-glycoside class of basic water-soluble substances (Yao et al., 2019). Mao et al. (2024) showed that 3% zhongshengmycin SL had a good effect on *Ophiopogon japonicus* root rot. In addition to root rot, zhongshengmycin SL has also shown a strong inhibitory effect on *Saccharum* tip rot (Liang et al., 2023) and *Canarium album* black spot (Shao et al., 2024) and is an efficient broad-spectrum fungicide.

In conclusion, the pathogen causing root rot disease in *A. crenata* Sims is *F. cugenangense*, with a lethal temperature of 59 °C. In the study, the most suppressive chemical pesticide was the 500 g/L Fluazinam SC, while the most suppressive biological pesticide was 3% zhongshengmycin SL. The results of this study provide a reference for identifying the cause of root rot in *A. crenata* Sims and for its control.

## Data Availability

The data that support the findings of this study are contained within the Supplementary material. All the sequences used in this study have been successfully uploaded to the NCBI repository. The nucleotide sequences obtained with TEF1, RPB1 and RPB2 have been uploaded successfully under the accession IDs (ZSG4: PQ476181, PX126198, PX108291; ZSG18: PQ476182, PX126199, PX108292; ZSG29: PQ476183, PX126200, PX108293); respectively.

## Footnotes

^1^ https://www.fusarium.org/
